# Variation in Cell Wall Metabolism and Flesh Firmness of Four Apple Cultivars during Fruit Development

**DOI:** 10.3390/foods11213518

**Published:** 2022-11-04

**Authors:** Qiufang Su, Xianglu Li, Lexing Wang, Bochen Wang, Yifeng Feng, Huijuan Yang, Zhengyang Zhao

**Affiliations:** 1 College of Horticulture, Northwest A & F University, Yangling 712100, China; 2 Apple Engineering and Technology Research Center of Shaanxi Province, Yangling 712100, China

**Keywords:** cell wall, softening and ripening, cultivars, development

## Abstract

Fruit ripening and softening are highly complex processes, and there is an interplay and coordination between the metabolic pathways that are involved in the biological processes. In this study, we aimed to elucidate the variation in the characters and possible causes of cell wall materials and morphological structure during apple fruits development. We studied the cell wall material (CWM), structure, cellular morphology, hydrolase activity, and the transcriptional levels of the related genes in four apple varieties ‘Ruixue’ and ‘Ruixianghong’ and their parents (‘Pink Lady’ and ‘Fuji’) during fruit development. The decrease in the contents of CWMs, sodium carbonate soluble pectin, hemicellulose, and cellulose were positively correlated with the decline in the hardness during the fruit development. In general, the activities of polygalacturonase, β-galactosidase, and cellulase enzymes increased during the late developmental period. As the fruit grew, the fruit cells of all of the cultivars gradually became larger, and the cell arrangement became more relaxed, the fruit cell walls became thinner, and the intercellular space became larger. In conclusion, the correlation analysis indicated that the up-regulation of the relative expression levels of ethylene synthesis and cell wall hydrolase genes enhanced the activity of the cell wall hydrolase, resulting in the degradation of the CWMs and the depolymerization of the cell wall structure, which affected the final firmness of the apple cultivars in the mature period.

## 1. Introduction

The quality of apple (*Malus domestica*) fruits is determined by a variety of the physiological, biochemical, and genetic events that alter the appearance, flavor, aroma, and texture during the fruit’s development and ripening [1]. Texture is an invaluable indicator of the intrinsic quality of apple fruits, which is usually evaluated by firmness, crispness, and juiciness [2]. Fruit firmness is an important intrinsic quality that affects a consumer’s choice, its processing, and its shelf life [3]. Apple fruits with a softer texture undergo mechanical damage and pathogen infections easily during transport and storage [4]. The softening of fruit involves multiple physical characteristics, including the disassembly of cell-wall polysaccharide, a reduction of the cellular turgor, and changes in the cuticle characteristics [5,6]. The precise mechanism of softening and which factor is more important have been the subject of decades of research, but they have remained elusive. As an economically important fruit quality character, increasing attention has been given to the research on the mechanisms that are involved in softening during development and ripening [7].

In addition to cell turgor pressure and the cuticle, the cell wall is the primary structure responsible for the textural characteristics of apple fruits [8]. The changes in fruit texture are mainly influenced by the dissolution of cell wall components and the remodeling of the cell wall structure [9]. The cell wall of the apple pulp is composed of three types of polysaccharides (pectin, hemicellulose, and cellulose) as well as small amounts of glycoprotein and water, which facilitate cell–cell adhesion, protect the cell, and maintain cell turgor [10]. Pectin, which is a key component of the middle lamella of cell wall, affected the physical properties and mechanical strength of the cell wall during growth [11]. The fruit firmness of diverse apple varieties, such as ‘Golden Delicious’, ‘Qinguan’, and ‘Fuji’, are positively correlated with the pectin levels [12]. Hemicellulose is composed of various cell wall materials (CWMs), such as xyloglucans, glucans, glucomannans, and xylans. They are often combined with cellulose microfibrils to provide a load-bearing molecular framework for the primary and secondary cell walls [6]. In general, cellulose microfibrils are embedded in long-chain xyloglucans through cross-bonding, and pectin fills the gaps between the cellulose/xyloglucan network to increase the integrity of the cell wall structure, thereby maintaining the hard texture of the apple fruit [13,14]. During apple fruit development, the apple’s firmness decreases with the cell wall polysaccharide remodeling and is eventually decomposed by multiple enzymatic complexes and non-enzymatic mechanisms [15].

The degradation of the cell wall polysaccharides involves the coordinated action of hydrolytic enzymes, such as polygalacturonase (PG), β-galactosidase (β-gal), pectate lyase (PL), pectin methylesterase (PME), xyloglucan-endotransglycosylase (XET), α-L-arabinofuranosidase (α-AF), and cellulase (CEL) [16]. Among the above enzymes, PG has been extensively studied and is considered to be the most effective hydrolase in the fruit ripening and softening processes [17]. A molecular marker of the PG gene *MdPG1* was discovered from the mapping of a quantitative trait locus (QTL) of post-harvest fruit firmness, which could explain up to 10.7% of the phenotypic variations [18]. A decreased apple pulp firmness is often accompanied with mealiness, to which *MdPG1* largely contributes [19]. The characteristics of the cell wall metabolism involved in the ripening and softening of various apple fruits are still not fully understood and have seldom been reported on. 

The process of fruit ripening requires the implication of many hormones, including gibberellins (GA), ethylene, and abscisic acid. Gibberellins plays an important role in fruit development, which are well correlated with cell division and cell expansion and can delay the ripening by regulating the transcript levels of the ethylene-related genes [20,21]. Ethylene is a phytohormone that is necessary for the ripening of climacteric fruits [22,23]. Ordinarily, the sudden production of ethylene can be detected during the early stages of apple fruit ripening, thus triggering a range of physiological changes, such as the decrease in hardness and brittleness [24]. Recently, it was found that QTL-based *MdACO1* and *MdACS1* markers, which have been used as molecular markers for the long-term shelf life of apple fruits, were associated with apple fruit hardness and their shelf life [25,26]. 

Efforts have been made to understand the physiological and biochemical changes that occur in apples during the softening process before and after their harvest, while most studies only used one variety [27]. ‘Ruixue’ and ‘Ruixianghong’ apples were selected from the same cross-combination as ‘Pink Lady’ and ‘Qinfu No. 1′. These two new late-ripening varieties are interesting for the market, and they have a good storability. We hypothesized that the reason for their storage tolerance might be closely related to their cell wall materials, structure, and them having a long developmental period. However, little is known about the regularity and mechanism of the firmness change that occurs in ‘Ruixue’ and ‘Ruixianghong’ apples and their parents during the development process. In this study, we studied the correlation between the fruit firmness and the alteration of CWMs, the hydrolase enzyme activity, and the structural characteristics during their development. Furthermore, we explored the expression patterns of some relative enzymes and ethylene biosynthesis genes during their development to clarify the change regularity and molecular mechanism of fruit firmness with the ripening of apple.

## 2. Materials and Methods

### 2.1. Plant Materials 

**Figure 1:**
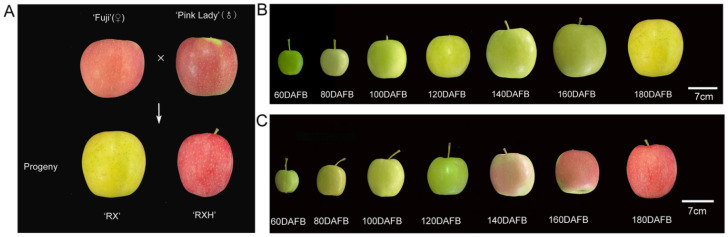
Appearance of different cultivars of apple in different fruit developmental stages. (**A**) Appearance of ‘Ruixue’ and ‘Ruixianghong’ and their parents apples. (**B**) Appearance of ‘Ruixue’ fruit at different development stages: 60 DAFB, 80 DAFB, 100 DAFB, 120 DAFB, 140 DAFB, 160 DAFB, and 180 DAFB. (**C**) Appearance of ‘Ruixianghong’ fruit at various development stages. DAFB: days after full bloom.

Apple fruits of four cultivars were collected from the experimental station of Northwest A&F University, Baishui County, Shaanxi Province, China. The ‘Ruixue’ and ‘Ruixianghong’ apples, as two new late-ripening varieties, were selected from the same cross-combination of ‘Pink Lady’ and ‘Qinfu No. 1′ (Figure 1A). The number of the ‘Ruixue’ cultivar is: CNA20151469.1. The four apple cultivars (all of them were eight years old) were grafted onto M26 rootstock and planted in 4.0 × 1.5 m plots. The management standard of the soil, water, fertilizer, pruning, and diseases, and pests in the orchards was consistent. The apples of the ‘Ruixue’, ‘Ruixianghong’, ‘Pink Lady’, and ‘Fuji’ varieties with uniform size and appearance and being without diseases, pests, and mechanical damage were selected at seven various developmental periods at 20-day intervals from 60 days after full bloom (DAFB) to maturity. At the point of maturity, Ruixue has a yellow-green skin, while the other three varieties have a red skin. Three biological replicates were used in the experiment. A total of 18 fruits were harvested, of which six fruits were considered as one biological repeat for every developmental period. All of the apple fruits were transported to the laboratory in a timely manner. Part of the fruits were used for physiology traits tests, and the pulp samples were cut into pieces, immediately frozen in liquid nitrogen, and stored in an ultra-cold storage freezer for a subsequent analysis.

### 2.2. Measurement of Physiological Characteristics

The firmness of the apple pulp was determined at two uniform locations of the equator at 90° of peeled fruits using a texture analyzer (GS-15, Germany; equipped with a 10 mm diameter flat probe with a test depth of 8 mm). The contents of sugar in various apple cultivars were detected by the method of Yang et al. [7]. The frozen flesh (2 g) was diluted to 20 mL with dd H_2_O, and extracted for an hour at 80 °C. After cooling it to ambient temperature, the supernatant was centrifuged at 10,000 r·min^−1^ for 10 min and then, it was filtered through a 0.22 μm filter membrane for performing high-performance liquid chromatography (HPLC). The total soluble sugar was calculated by the summing the fructose, sucrose, glucose, and sorbitol values. The titratable acidity of the apple fruit was measured using a fruit acidity meter (GMK-835F Perfect, Berlin, Germany). 

### 2.3. Analysis of CWMs

The cell wall polysaccharide was extracted according to the method of Melton and Smith with some modifications [28]. In short, the frozen sample (3.0 g) was used to extract the CWMs by acetone and dimethylsulfoxide. From the obtained CMWs, (1, 2-cyclohexylenedinitrilo)-tetra acetic acid (CDTA, 0.05 M), Na_2_CO_3_ (0.05 M), and KOH (4 M) were used sequentially to extract the distinct types of pectin and hemicellulose polysaccharides. The resulting cell wall residue was dominated by cellulose. 

The contents of pectin and hemicellulose were measured by the method of carbazol-ethanol and anthrone-sulfuric acid method, and the unit of measurement was mg·g^−1^ fresh weight (FW). 

### 2.4. Determination of Hydrolytic Enzyme Activity

The hydrolytic enzymes of the cell wall were assayed according to the methods described by Brummell et al. [29]. The extraction of three enzymes was performed using the method of Ge et al. [30]. The flesh tissues (10.0 g) were powered in liquid nitrogen using a cooling mortar, and then, extracted with 20.0 mL 95% ethanol for 20 min at 4°C. After the homogenate was centrifuged at 4 °C for 20 min (12,000× *g*), the pellet was washed three times with 10.0 mL of ice-cold 80% ethanol. Finally, the pellets were extracted with 5 mL of 50 mmol L^−1^ sodium acetate buffer (pH 5.5, containing 1.8 mol L^−1^ NaCl) to measure the PG, β-gal, and CEL contents. The PG and cellulase enzyme activity was detected by the dinitrosalicylic acid colorimetric method as described in Chen et al. [31]. For the PG activity, 0.5 mL crude enzyme was added to 1.0 mL sodium-acetate (pH 5.5, 50 mM) and 0.5 mL of 10 gL^−1^ polygalacturonic acid. After 1 h of incubation at 37 °C, the mixture was mixed with 1.5 mL 3,5-dinitrosalicylic acid (DNS) and then, it was boiled for 5 min. After cooling, we added distilled water to 25 mL of the mixture, and then, the absorbance was measured at 540 nm. The cellulase activity was determined in a similar way to that for the PG. The activity of β-gal enzyme was represented as the release of µmol p-nitrophenyl-β-D-galactopyranoside (PNP) min^−1^·g^−1^ FW using the method of Li et al. with a minor modification [32]. It was performed in a mixture including 0.5 mL of 10 g/L PNP and 0.5 mL crude enzyme. Then, it was incubated at 37 °C for 30 min, which was followed by the addition of 2 mL of 1 mol/L Na_2_CO_3_. The concentration determined by its absorbance at 400 nm.

### 2.5. The Microstructure of Fruit Pulp Cells

The microstructure of the apple pulp was observed by using a scanning electron microscope (SEM) according to the method described by Wang et al. [33]. Briefly, the pulp at the equator (5 × 3 × 2 mm) was fixed with 4% glutaraldehyde over six hours, and then, it was washed more than five times with phosphate-buffered saline (PBS, pH 6.8), which was followed with a series of graded alcohol (10–100%) for 10 min and 25, 50, 75, and 100% tert-butanol for 15 min each, respectively. Afterwards, the sample was freeze-dried for about three hours. Then, it was glued to the worktable, gilded, and observed at 5 kV using a S-3400N SEM (FEI Quanta 200, Thermo Fisher Scientific, Bedford, MA, USA).

### 2.6. Morphology Analysis of Apple Fruit Cell Wall 

The morphology of the apple pulp cell wall was analyzed by using transmission electron microscope (TEM) according to the method described by Li et al. [34]. Small cuboids at the equator of the apple flesh (5 × 2 × 1 mm) were cut, fixed with 4% glutaraldehyde at 4 °C overnight, washed over three times by PBS, and fixed in 1% osmium tetroxide for 2 h. Then, they were washed five times with PBS again. After their dehydration using a series of graded ethanol, the samples were saturated in LR White resin at 55 °C for more than two days. Finally, ultrathin sections (70 nm in thickness) were cut using an ultramicrotome (Ultracut-R, Leica, Germany) and were viewed under a TEM (JEM 1230, JEOL, Japan). 

### 2.7. Quantitative Reverse Transcription Polymerase Chain Reaction (RT-qPCR) 

The total RNA concentration of the apple pulp samples were extracted through a Quick RNA isolation kit (Tiangen, Beijing, China), and the cDNA reverse transcription was performed using the All-in-One First-Strand cDNA Synthesis SuperMix with gDNA Eraser (TransGen Biotech, Beijing, China). Using the ABI7500 System and SYBR Green Master Mix (Vazyme, Nanjing, China), a RT-qPCR was performed. Actin served as an internal control. The primers for the RT-qPCR are shown in Table S1. The genebank accession numbers are shown in Table S2.

### 2.8. Statistical Analysis

The data analysis of the difference was conducted using a one-way ANOVA with Duncan’s test. *p* < 0.05 indicates a statistically significant difference. The statistical calculations were analyzed using the SPSS (19.0, SPSS Inc., Chicago, IL) software.

## 3. Results

### 3.1. Physiological Characteristics of ‘Ruixue’ and ‘Ruixianghong’ and Their Parents Apples during Fruit Development

**Figure 2:**
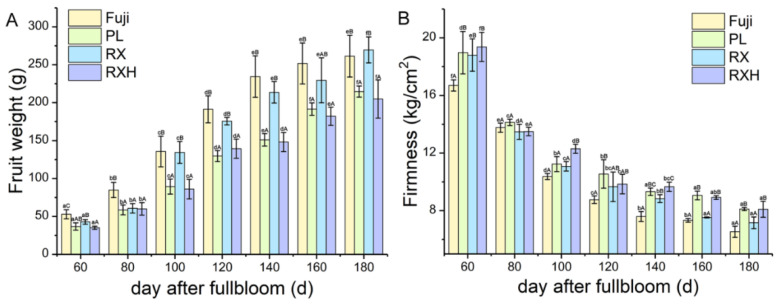
The variation of physiological characters in the ‘Ruixue’ and ‘Ruixianghong’ varieties and their parents apples during fruit development. (**A**) Fruit weight; (**B**) fruit firmness. Different lowercase letters in columns denote significant differences between sampling dates for each cultivar by Duncan’s multiple range test (*p* < 0.05). Different capital letters in columns denote significant differences by Duncan’s multiple range test (*p* < 0.05) among different cultivars. Different colors represent different cultivars.

A total of seven developmental periods were analyzed at 20-day intervals throughout the development. As is shown in Figure 1B,C, the skin color of the ‘Ruixue’ apples changed from green to yellow, while the peel color of the ‘Ruixianghong’ apples changed from green to red. In terms of the growth during the process of their development, the ‘Ruixue’ apples showed the characteristics of inheritance from its parent ‘Fuji’ in terms of its size, while the ‘Ruixianghong’ apples showed a trend similar to that of the ‘Pink Lady’ one (Figure 2A). At 160 DAFB, the firmness of the ‘Pink Lady’ and ‘Ruixianghong’ ones were significantly higher than those of the ‘Fuji’ and ‘Ruixue’ ones, and a similar trend was maintained thereafter (Figure 2B). From 60 DAFB to the mature period, the contents of fructose and sucrose showed an increasing trend in the four apple cultivars during the developmental stage, reaching a peak at 180 DAFB. When it was 60 DAFB, the sucrose content was lower than that of glucose content in the four cultivars. However, as the fruit matured, the sucrose content was higher than that of glucose content in the same period (Figure S1). Overall, in the four apple varieties, the total soluble sugar in the developmental period showed an overall increasing trend (Figure S2A). Otherwise, the titratable acids of the four cultivars showed a trend of declining during the developmental period. During the 60–180 DAFB period, the titratable acid content of the ‘Pink Lady’ type was significantly higher than those of the other three varieties ( Figure S2B).

### 3.2. Measurement of CWMs in the Four Apple Cultivars during Development

**Figure 3:**
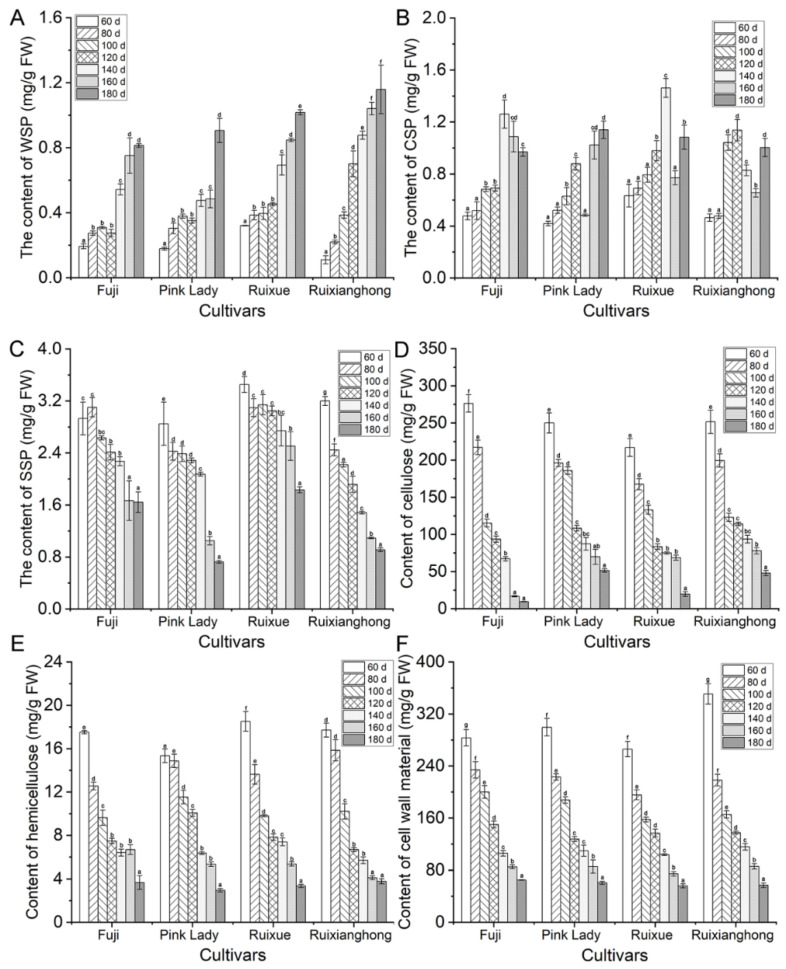
The alteration of cell wall polysaccharides in the ‘Ruixue’ and ‘Ruixianghong’ varieties and their parents apples during fruit development and ripening. (**A**) The content of water-soluble pectins (WSP), (**B**) chelator soluble pectins (CSP), (**C**) sodium carbonate soluble pectins (SSP), (**D**) cellulose, (**E**) hemicellulose, and (**F**) cell wall material. Different letters indicate significant differences between sampling dates for each cultivar (*p* < 0.05).

The loss of the pulp’s hardness is often associated with a decline in the cell turgor pressure with the metabolism in the cell wall [1]. In our study, water-soluble pectins (WSP), chelator soluble pectins (CSP), and sodium carbonate soluble pectins (SSP) were determined. The WSP content of the four apple cultivars showed an overall increasing trend from 60 DAFB to the maturity stage (Figure 3A). The CSP content of the four apple varieties showed a trend of first, rising and falling, and then, rising again, apart from in the ‘Fuji’ one (Figure 3B). However, for the SSP, there was a declining trend in the four varieties (Figure 3C), and the cellulose and hemicellulose contents of the four apple varieties exhibited similar declining trends at all of the developmental stages (Figure 3D,E).

The apple fruits’ CWMs were measured in the four cultivars from 60 DAFB to maturity. The content of CWMs showed a similar declining trend in the four apple varieties during the whole development period. From the fruitlet stage (before 60 DAFB) to the expansion stage (from 80–140 DAFB), the decline was faster, but the decline was slower from the expansion to the maturity stage (140–180 DAFB) (Figure 3F). 

### 3.3. Measurement of Cell Wall Degrading Enzymes Activity in the Four Apple Cultivars during Development

**Figure 4:**
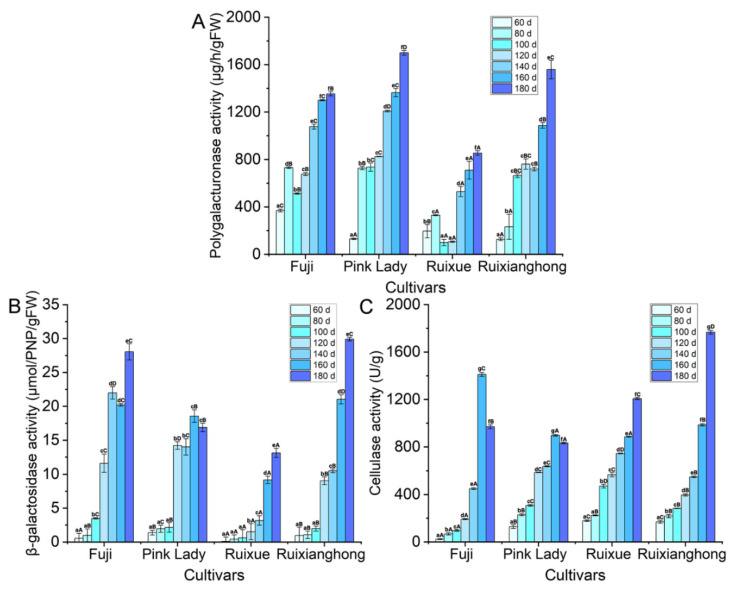
Changes of cell wall degrading enzymes activity in the ‘Ruixue’ and ‘Ruixianghong’ varieties and their parents apples during fruit development and ripening. (**A**) Polygalacturonase (PG), (**B**) β-galactosidase (β-gal), and (**C**) cellulase (CEL). Data are presented as means from three independent experiments; the vertical bars indicate the standard error of the mean. Different lowercase letters in columns denote significant differences between sampling dates for each cultivar by Duncan’s multiple range test (*p* < 0.05). Different capital letters in columns denote significant differences by Duncan’s multiple range test (*p* < 0.05) among different cultivars. Different colors represent different periods.

The depolymerization and dissolution of pectin and hemicellulose polysaccharides in the cell wall are the major processes of fruit softening [9], so the key cell wall hydrolases such as PG, β-gal, and CEL activity were determined. At the later stages of development, especially between 140–180 DAFB, the PG enzyme activity of the four cultivars was significantly enhanced (Figure 4A). At the point of maturity, the PG enzyme activity of ‘Ruixue’ variety was significantly lower than those in the remaining varieties. The activities of β-gal were similar in the four apple varieties at the fruitlet stage, but they increased noticeably at the expansion and mature fruit stages. Furthermore, in the period of 180 DAFB, the β-gal enzyme activity of the ‘Ruixianghong’ type was the highest among all of the cultivars, and the β-gal enzyme activity of the ‘Ruixue’ type was significantly lower than those of the other three cultivars (Figure 4B). The cellulase enzyme production showed an increasing trend in the four apple cultivars during the apple fruit development (Figure 4C). In particular, the degradation of SSP by the β-gal and PG enzymes may affect the cell wall structure in the apple fruit. In brief, the decrease in the CWMs may be due to the increased activity of cell wall hydrolysis, which affects the final firmness at the point of maturity of the apple cultivars.

### 3.4. Microstructure Analysis in the Four Apple Cultivars during Development

**Figure 5:**
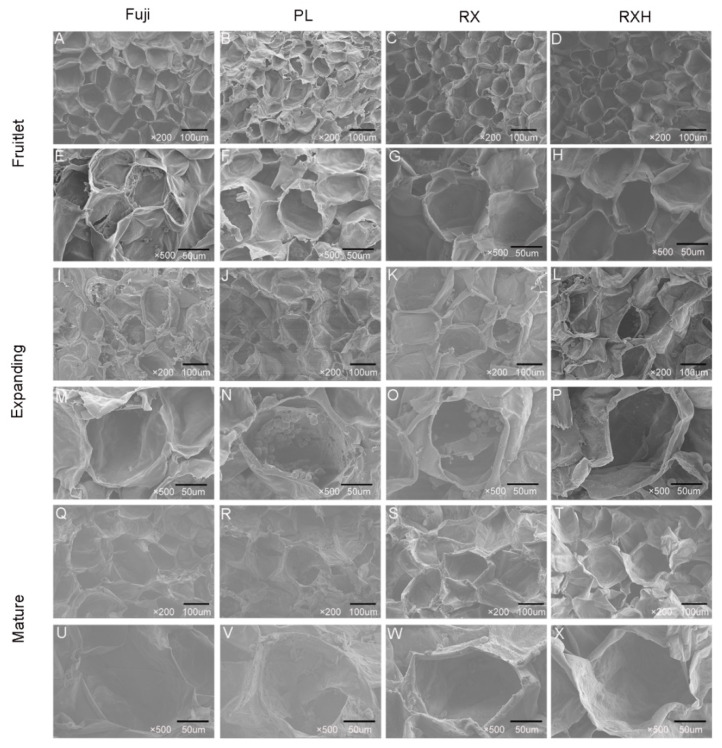
Scanning electron micrograms showing tissue fracture patterns during ripening progress of different cultivars of apple fruit. (**A**–**D**,**I**–**L**,**Q**–**T**) were obtained at 200× magnification (bar, 100 mm), and (**E**–**H**,**M**–**P**,**U**–**X**) were obtained at 500× magnification (bar, 50 μm).

Fruit texture is largely related to cell structure and morphology [2]. In order to explore the regularity in the changes to the apple fruit firmness, the microstructure of the apple flesh tissue at the three distinct developmental stages was observed by using SEM. From the fruitlet to the mature stage, as the fruit grows, the fruit parenchyma cells of all of the four cultivars gradually became larger, the cell arrangement became more relaxed, the fruit cell walls became thinner, and the intercellular space became larger than it was in the young fruit stage (Figure 5). In the fruitlet stage, all of four cultivars displayed a uniform, complete, and clear honeycomb structure. The cells were arranged neatly and tightly, intracellular breakages were observed, and the gaps between the adjacent cells were minimal (Figure 5A–D). In addition, the fractured surfaces of the fruit cells of the ‘Fuji’, ‘Ruixue’, and ‘Ruixianghong’ types were relatively smoother, more flat and cleaner in the fruitlet stage. However, the fractured surfaces of the ‘Pink Lady’ fruit cells was relatively rough and uneven, with it having hairy filaments at the same stage (Figure 5B,F). During the middle and later stages of development, the differences in the fractured surfaces between the cultivars tended to disappear gradually. No significant differences in the fractured surfaces between these varieties at the mature stage were observed, and the cell fracture of the four late ripening varieties were more likely to occur on the equatorial line. However, the size and arrangement of the apple fruit cells and the cell wall thickness affected the texture changes during the development.

### 3.5. Ultrastructural Analysis of the Four Apple Cultivars during the Mature Stage 

**Figure 6:**
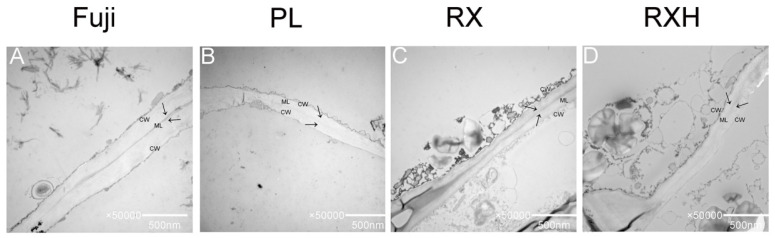
Transmission electron microscope of different cultivars during the fruit ripening stage. (**A**) Fuji; (**B**) Pink Lady; (**C**) Ruixue; (**D**) Ruixianghong. CW: cell wall, ML: middle lamella. (**A**–**D**): 50,000×; size bars, 500 nm.

The structure and morphology of the cell wall largely contributes to the fruit’s texture (firmness and brittleness). In order to understand the characteristics of the structures of the cell wall of different apple cultivars at the mature stage, the cell wall pulp of the above varieties were observed using TEM at the mature stages. Pectin is the major component of the middle lamella in the cell wall, which influences cell wall rigidity and adhesion, and the middle lamella of these apple cultivars showed some distinction at the point of maturity. As shown in the picture, the adjacent areas of the cell walls of all of the four cultivars exhibited broad light–dark–light bands, which indicates that the cell wall structure remained intact, and the fibrous material was tightly packed. Compared to those of the ‘Ruixue’ and ‘Fuji’ type, they showed a severe reduction in the dark regions of the ripening fruits of the ‘Pink Lady’ and ‘Ruixianghong’ type. The differences between the four varieties was in the area of the middle lamella, which appeared to be dispersed in the ‘Pink Lady’ type (Figure 6). 

### 3.6. Expression of Cell Wall and Ethylene-Related Genes in the Four Apple Cultivars during Development 

**Figure 7:**
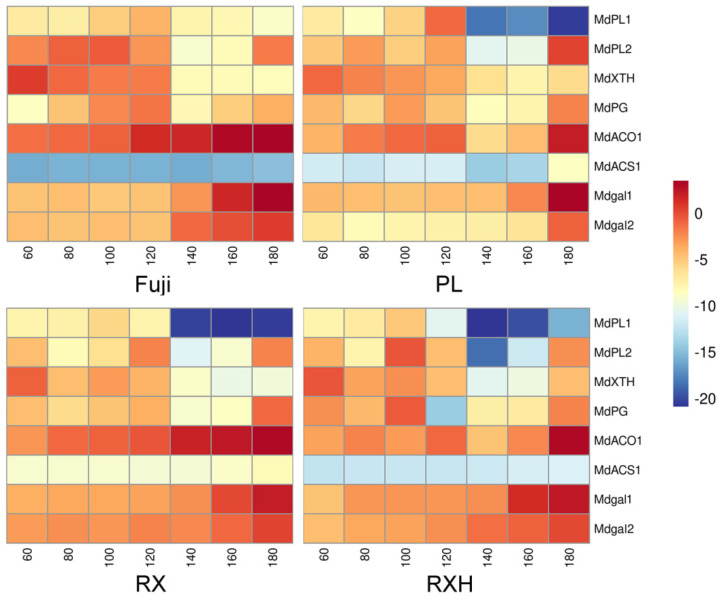
Expression heatmap of ethylene synthesis and cell wall hydrolytic-related genes for hardness decline during fruit development.

From our previous transcriptomic data [27], we screened the cell wall hydrolase and ethylene related genes, whose expression levels altered significantly during the fruit development. The relative expressions of eight genes were detected in the distinct apple cultivars, and the developmental periods were detected by the qRT-PCR (Figure 7). At the distinct fruit developmental stages, eight genes showed various expression patterns as follows: the relative expression level of MdPL1 increased from low to peak values and then, decreased during the whole developmental phase. Moreover, it reached the maximum expression level at 100 DAFB for the ‘Pink Lady’, ‘Ruixue’, and ‘Ruixianghong’ type, while the ‘Fuji’ one peaked at 140 DAFB. The expression trends of MdPL2 and MdPG during the developmental period are similar, with a trend to first increase and then, decrease from 80 to 140 DAFB; it shows a rapid increase at the later developmental stages in the four cultivars. However, the rising trend of the ‘Fuji’ and ‘Ruixianghong’ types is obviously higher than those of the other two varieties at this expansion stage. In addition, the relative expression of MdXTH decreased rapidly during the developmental phase of the four cultivars, whose expression level was higher in the fruitlet stage in all of the cultivars. Moreover, the expression patterns of two β-gal genes were markedly upregulated in the later stages of development in the four cultivars. Notably, the expression pattern of the Mdβ-gal2 gene was upregulated to a higher degree in the ‘Fuji’ one than it was in the ‘Ruixue’, ‘Ruixianghong’, and ‘Pink Lady’ ones. The expression of MdACO1 and MdACS1 were greatly increased at the later developmental stages, except for the expression of MdACS1 in the ‘Ruixianghong’ and ‘Fuji’ types. 

## 4. Discussion

### 4.1. Dynamic Variations of Physiological Characteristics of ‘Ruixue’ and ‘Ruixianghong’ Apples and Their Parent Apples during Fruit Development

Fruit firmness is a key trait that determines the fruit’s transportation and storage, which is the primary goal of breeding [6]. Exploring the cytological and molecular mechanisms that regulate fruit firmness has crucial theoretical significance for assisting the cultivation of high-quality apple varieties. Many studies have described the involvement of the cell walls in fruit ripening and softening in various species, including tomatoes [35], bananas [36], peaches [37], pears [38], and apples [39], while most studies have used only one variety of them. In this study, we dissected the regulatory mechanisms that are involved in the ripening and softening of the cell wall components and structures of new late-ripening apple cultivars during their development.

At present, the selection of parents is very critical in cross-breeding. In our experimental materials, using the ‘Fuji’ and ‘Pink Lady’ types as parents, two new cultivars of ‘Ruixue’ and ‘Ruixianghong’ were selected. During their maturation, the firmness of the ’Ruixianghong’ type seemed to be similar to that of the firm ‘Pink Lady’ type, while that of the ‘Ruixue’ type was similar to that of the ‘Fuji’ one. Moreover, both the ‘Pink Lady’ and ‘Ruixianghong’ types were firmer than the other two varieties. In addition, the ‘Ruixue’ one fully inherited the large fruit characteristics of the ‘Fuji’ one in terms of the fruit weight, while the ‘Ruixianghong’ one was very similar to the ‘Pink Lady’ one in terms of size. These results further confirmed that the ‘Pink Lady’ and ‘Fuji’ types were the core parent materials for breeding.

### 4.2. Analysis of Changes in Cell Wall Components during Development in Four Cultivars

The remodeling and metabolism of the cell wall materials contribute significantly to the changes in the apple’s texture [8]. Pectin substances (WSP, CSP, and SSP), hemicellulose, and cellulose are the major components of the cell wall polysaccharides, the biochemical degradation of which is closely related to the decrease in the intercellular adhesion and the cell wall disintegration as the softening process progresses [32]. The dissolution of insoluble protopectin is the most fundamental characteristic of fruit softening. In our study, it showed that with fruit development, the WSP content showed an increasing trend in the four cultivars, whereas the CSP content first showed an increasing trend, which was followed by a declining one, and finally a raising trend again. This is possible because protopectin was degraded at the early stages of the development, and then, pectin was converted into pectinic acid. In the final mature stage, the methylated pectin was converted into water-soluble pectin under the action of pectin-related degrading enzymes. In all of the four apple varieties, the SSP content decreased with the fruits’ ripening, which was consistent with previous reports [40,41]. In addition, the cellulose, hemicellulose, and total CWMs also decreased continuously, which was expected during the fruits’ growth. Therefore, the reduction in the fruit firmness may be highly correlated with the degradation of SSP, cellulose, and hemicellulose in this study.

### 4.3. The Impact of Cell Wall Hydrolase of Softening on the Various Apple Cultivars during the Ripening Process

Cell wall hydrolase enzymes speed up the breakdown of the cell wall materials such as pectin, cellulose, and hemicellulose, thereby causing the ripening and softening of fruit [9,29]. Pectin, the most complex polysaccharide in the cell wall, fills the gap between the cellulose and hemicellulose networks and plays a vital role in the change to the fruits’ texture, the degradation of which is completed through the synergistic functions of multiple enzymes. Previous studies have shown that the PL, PG, β-gal, PME, and α-AF enzymes could degrade pectin and thus, affect the integrity of the cell wall [42]. Our study focused on the effect of two key pectin-degrading enzymes (PG and β-gal) on the fruit ripening process [39]. It was reported that PG enzyme activity is largely responsible for the ripening and softening of apple fruits [17]. The elevation in the PG enzyme activity of the four cultivars at mature phases supports the hypothesis that it might contribute to reducing the fruit firmness by depolymerizing homogalacturonan (HG) in pectin [18,43]. It is well known that β-gal is a highly active enzyme that is commonly found in an apple’s development, especially during ripening [4]. This study showed that the activity of β-gal increased markedly with the fruits’ growth, especially in the later stages of their development, which might have strengthened the degradation of protopectin, thereby leading to the fruit cell wall becoming loosened and ultimately reducing the firmness of the apple fruit. Cellulose is also the major component in cell walls, and CEL is a kind of cellulose-degrading hydrolase [44]. In our study, a dramatic increase in the CEL activities of the four cultivars was observed during their development, indicating that it was negatively correlated with the change in hardness of the four cultivars.

### 4.4. Microstructural Alterations of Fruit Firmness during Development in the Four Cultivars

Previous studies found that cell size and shape, cell wall thickness, and turgor pressure were major factors that affected the fruits’ firmness [13]. Previous research has shown that firmer varieties have tighter cell arrangements and smaller intercellular spaces than those of other varieties of similar crispness [32]. However, the cell arrangements and pulp cellular gaps in those cultivars were similar during the maturation stages (Figure 5Q–T). The possible reason for this was that the cell morphology was not the major factor determining the firmness during this period in different apple cultivars. Allan-Wojtas’s research showed that the apple varieties with crisp textures had more neat and clean fractured cell surfaces [45]. Our results showed that there was a certain difference in the fractured surfaces of the apple fruit cells of these varieties at the fruitlet stage, and this difference became smaller and finally, it disappeared as the fruit developed. In addition, all of the apple parenchyma cells of various cultivars exhibited a regular arrangement with a good texture during maturation, suggesting that the ‘Ruixue’ and ‘Ruixianghong’ apples inherited the excellent texture of their parents.

### 4.5. Cell Wall Ultrastructure in the Different Apple Cultivars at Maturity

The softening of the fruit during ripening may be due to a series of modifications in the polymer network that makes up the cell walls, leading to a decrease in the fruit’s hardness during its development. Pectin is a key component in the middle lamella of the cell wall, and the decline in the cohesion of cellular network is the main reason for the softening of many fruits during the mature stage [46]. By observing the cell wall structure of different varieties, it was found that compared to that of the other varieties, the middle lamella of the ‘Ruixue’ and ‘Fuji’ fruit was brighter and clearer than the other two varieties, which might have a longer shelf life. Previous research reported that ‘Honeycrisp’ apple fruit kept its cell walls intact after six months of post-harvest storage, while the cell walls of its parents deteriorated [47]. It was also reported that the cell wall of the ‘Ruixue’ apple can still be maintained for a few months after its harvest [34]. Moreover, the specific mechanism of its storability still requires further investigation. For four late-maturing apple cultivars, the long growth period might help to form a highly solidified and a strongly cross-linked cell wall network, thus, preventing the modification of cell wall morphology, which was beneficial to avoid the alteration of the texture during the mature stage [48].

### 4.6. Analysis of Expression Patterns of Ethylene Synthesis and Hydrolysis Enzyme-Related Genes during Fruit Development Involved in Cell Wall Metabolism Pathways

By studying the detailed expression patterns of genes related to the regulation of cell wall polysaccharides during the developmental process in various apple cultivars, we could gain insight into the mechanisms that affect apple firmness properties and screen new genes that are involved in these processes [1]. The study of the key gene expression patterns of cell wall hydrolases was of great significance for further understanding the molecular basis of the fruit ripening process. Through a qPCR, we found that the relative expression of *MdPL1* changed from low to high and then, it decreased. In contrast, the relative expression of *MdXTH* decreased significantly and *MdPG* increased significantly, indicating that the PG and PL enzymes might have a significant positive correlation with the degradation of pectin. Many studies have shown that the PG genes play a crucial role in the rapid softening of pears [38], grapefruits [49], and apples [50]. It has also been shown that hemicelluloses undergo main structural changes, especially during early fruit development [1], so *MdXTH* might transport or hydrolyze xyloglucan in the early stage of the apple fruit’s development. In addition, the relative expressions of two β-gal candidate genes were upregulated to varying degrees in the later developmental stages of the four varieties, indicating that their effect was inconsistent, which might result in different firmness properties in the mature stages. It has also been reported that there was a ripening-specific β-gal gene, and the downregulation of *TBG4* expression inhibited the fruit softening process in tomatoes [51].

Previous studies have demonstrated that ACO and ACS enzymes are involved in the production of ethylene and serve as marker genes for fruit ripening [52]. The transcriptional levels of *MdACO1* and *MdACS1* were significantly upregulated when they reached the ripening stage, which was an important signal of ethylene production in apple fruits. As a marker gene for the post-harvest storage period of fruit, it indicated that a small amount of ethylene might be synthesized when the fruit was close to ripening. It took approximately a month after harvest to reach its peak respiration [53], and the variations in the transcript levels of the cell wall-related genes have been analyzed between distinct apple varieties, supporting the notion that fruit ripening and softening depended on genetic regulation processes [54,55].

## 5. Conclusions

This study could provide informative physiological, biochemical, structural, and molecular evidence for the apple fruits softening of new cultivars during the development stage. With the development of the fruit, the activity of cell wall hydrolase and the relative expression levels of the related genes were enhanced by regulating the cell wall materials’ degradation, and these results demonstrated that the decline of the firmness of the new cultivars in apple fruit during their development and ripening are determined, at least in part, by the control of cell wall materials’ dissolution and the modification to the structure. At the mature stage, the cell fracture of the four late ripening varieties were more likely to occur on the equatorial line, which indicated that the two new late-ripening varieties inherited the excellent texture from their parents.

## Data Availability

The datasets generated for this study are available on request to the corresponding author.

## Supplementary Materials

The following supporting information can be downloaded at: https://www.mdpi.com/article/10.3390/foods11213518/s1, Figure S1: The variation of sugar content in the ‘Ruixue’ and ‘Ruixianghong’ varieties and their parents apples during fruit development; Figure S2: The variation of total soluble sugar and titratable acid content in the ‘Ruixue’ and ‘Ruixianghong’ varieties and their parents apples during fruit development. Table S1: The primers used for qRT-PCR; Table S2: The genebank accession numbers used for qRT-PCR.
