# Supplementary material for: Variation in Cell Wall Metabolism and Flesh Firmness of Four Apple Cultivars during Fruit Development
Source: Foods. 2022 Nov 4;11(21):3518. doi: 10.3390/foods11213518 (PMC9656455; doi:10.3390/foods11213518)
Supplement: Supplementary file 1 [file foods-11-03518-s001.zip › foods-1962538-supplementary.pdf]

Supplementary Material

Qiufang Su, Xianglu Li, Lexing Wang, Bochen Wang, Yifeng Feng, Huijuan Yang, Zhengyang Zhao*

***Correspondence: Zhengyang Zhao**

Table S1. The primers used for qRT-PCR

Primer name	Forward primer	Reverse primer
MdPL1-qPCR	CTTACGGGGGAGGA GGGTG	TGGGCAATGTGCTG GAAGA
MdPL2-qPCR	CATCCTGTAAACCCT AAACCCG	CTACCACCAAAAAT GGAGACCC
MdXTH-qPCR	ACAGTCTGGCTTGAT AGCACCAC	AACACTTTTCCTAGC ATACCTCC
MdPG-qPCR	CGACTTCGCCACCAC CGT	CCGAGCCCAAAGCC ATAAA
MdACO1-qPCR	GACTTGGACTGGGA AAGCAC	GGAGGGTAGTTGCT GACCTT
MdACS1-qPCR	AGCCTCTCTAAGGAT CTTGG	TGGTTCTCGGCTATG TAGTTCTT
MdActin-qPCR	CTTCAATGTGCCTGC CATGTAT	AATTTCCCGTTCAGC AGTAGTG
Mdβ-gal1-qPCR	GGGAGTTCTTCTGTT GAATGGG	TTCCGGCATAAGAA CAATCG
Mdβ-gal2-qPCR	CGAAATACCCTGTTA GAGTGAGC	CGATGAATGACTGC CAAGGAA

Table S2. The genebank accession numbers used for RT-qPCR

MdPL1	MD16G1070600
MdPL2	MD16G1106400
MdPG	MD16G1092600
MdXTH	MD12G1109400
MdACO1	MD10G1328100
MdACS1	MD15G1302200
Mdβ-gal1	MD02G1079200
Mdβ-gal2	MD15G1206800

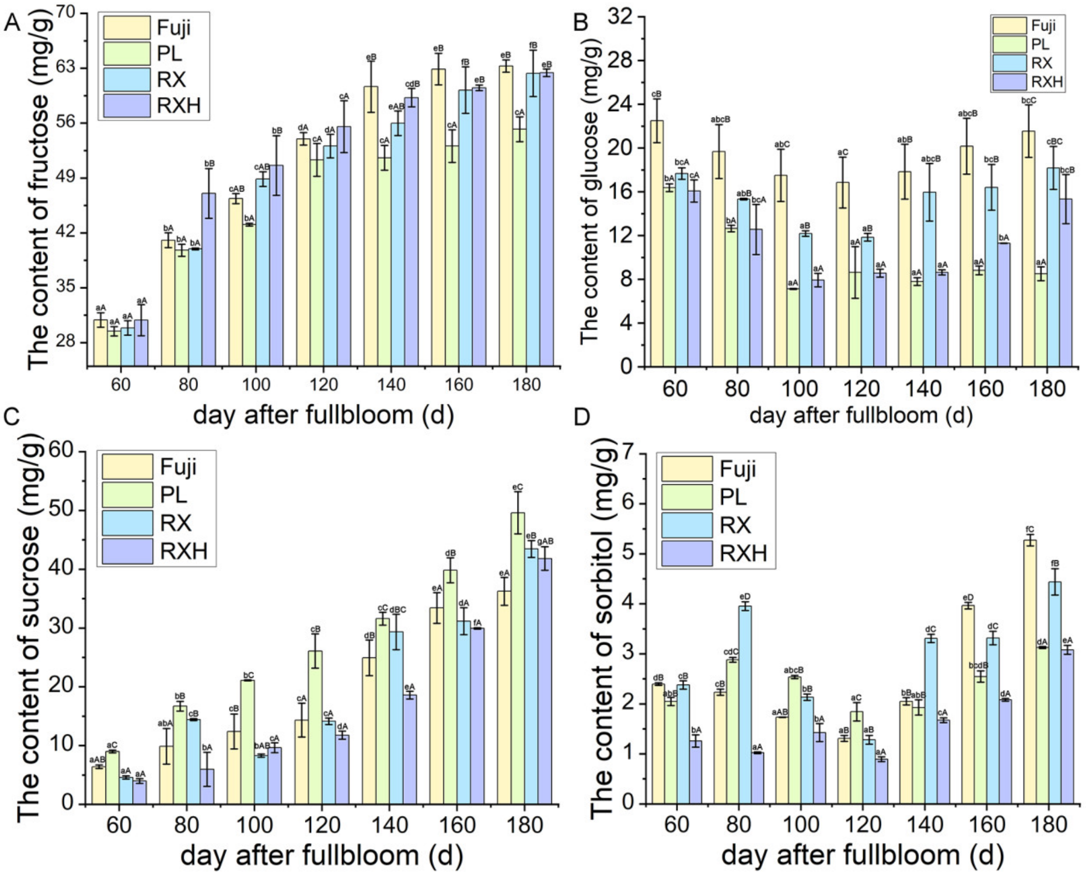

Figure S1. The variation of sugar content in 'Ruixue' and 'Ruixianghong' and their parents apples during fruit development. (**A**) Fruotose (**B**) Glucose (**C**) Sucrose (**D**) Sorbitol DAFB: days after full bloom. Different lowercase letters in columns denote significant differences between sampling dates for each cultivar by Duncan's multiple range test ($p < 0.05$). Different capital letters in columns denote significant differences by Duncan's multiple range test ($p < 0.05$) among different cultivars. Different colors represent different cultivars.

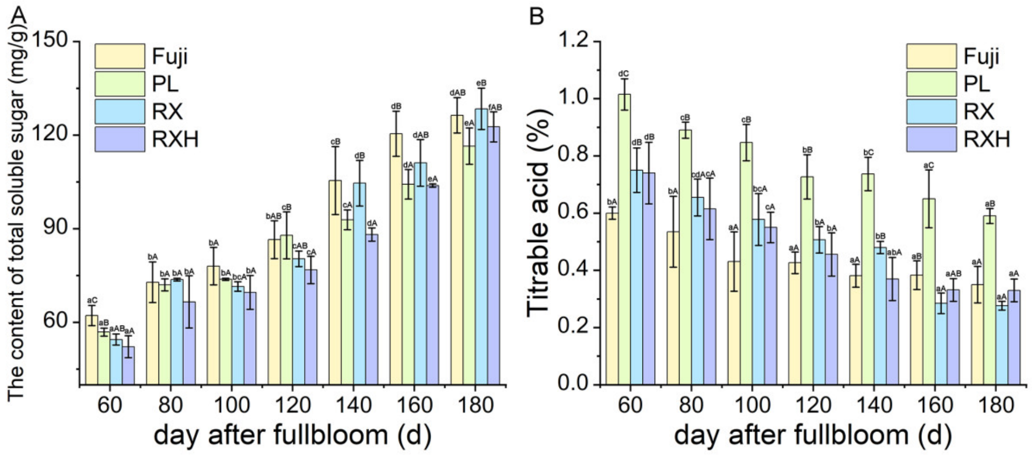

Figure S2. The variation of total soluble sugar and titrable acid content in 'Ruixue' and 'Ruixianghong' and their parents apples during fruit development. Different lowercase letters in columns denote significant differences between sampling dates for each cultivar by

Duncan's multiple range test ($p < 0.05$). Different capital letters in columns denote significant differences by Duncan's multiple range test ($p < 0.05$) among different cultivars. Different colors represent different cultivars.
